# CTLA-4 methylation regulates the pathogenesis of myasthenia gravis and the expression of related cytokines

**DOI:** 10.1097/MD.0000000000010620

**Published:** 2018-05-04

**Authors:** Ti-Kun Fang, Cheng-Jun Yan, Juan Du

**Affiliations:** Department of Emergency, Jining No.1 People's Hospital, Jining, Shandong Province, P.R. China.

**Keywords:** cytokine, cytotoxic T lymphocyte correlated antigen-4, methylation, myasthenia gravis

## Abstract

**Background:**

Myasthenia gravis (MG) is a progressive autoimmune disease that occurs as a result of the failure of neuromuscular transmission and is characterized by muscle weakness. There has been evidence on the correlations between the genetic predisposition of cytotoxic T lymphocyte and the antigen-4 (CTLA-4) and MG. Thus, the present study was conducted to study is designed to examine the effects of CTLA-4 methylation on the pathogenesis of MG and the expressions of related cytokines.

**Methods:**

The CTLA-4 methylation levels in peripheral blood were quantified in 103 samples collected from MG patients and 86 samples from healthy individiuals. The expression of serum-related cytokines as well as the Treg cell ratio were examined so as to define the contributory role of CTLA-4 methylation in MG and to identify the interaction between CTLA-4 methylation and related factors, the expressions of DNA methyltransferase (DNMT)l, DNMT3A and DNMT3B, CTLA-4, AchR-Ab, Titin-Ab, RyR-Ab, IL-2, IL-10, IFN-γ, and TGF-β, activity of P- acetylcholinesterase (AchE) and E-AchE.

**Results:**

The results indicated that the incidence of CTLA-4 methylation was significantly higher in the control group when compared with the MG group, and CTLA-4 methylation was also found to be associated with the thymus status of MG patients. It was also observed from the experiment data that the expressions of DNMTl, DNMT3A, and DNMT3B, along with the expressions of AchR-Ab, Titin-Ab, RyR-Ab, IL-2, IL-10, IFN-γ and TGF-β, and the activity of P-AchE and E-AchE were all higher in the MG group than in the control group, with a reduction of CTLA-4 expression. Another key finding from this study revealed that methylation interference can lead to the suppression in the expression of AchR-Ab, the activity of E-AchE, the expression of IL-2, IL-10, IFN-γ, and TGF-β and the Treg cell ratio in lymphocytes.

**Conclusion:**

In conclusion, the results obtained from the present study highly indicated that CTLA-4 methylation might play a role in facilitating the occurrence of MG and increasing the expressions of related cytokines through the upregulation of AchR-Ab and E-Ach.

## 1 Introduction

Myasthenia gravis (MG) is an autoimmune disease of the nervous system with autoantibodies against acetylcholine receptors at the neuromuscular junction, and characterized by muscle weakness with fatigue.^[1]^ Based on the clinical manifestations, MG is typically divided into ocular MG and generalized MG. Although the effect of ocular MG is only on the extraocular muscles, generalized MG affects all kinds of muscles in the body, including bulbar, facial, respiratory, and limb muscles.^[2]^ The incidence of MG is nearly 30 per 1 million per year and its prevalence is estimated to be 25 to 142 per million.^[3]^ MG is clinically characterized by fatigue and weakness in the ocular, bulbar dysarthria, dysphagia or limb muscles: in severe cases, MG can lead to death from breathing difficulties.^[4,5]^ The treatments for this disease include the use of anticholinesterase drugs and immunotherapy in order to improve neuromuscular transmission.^[6,7]^ Based on recent studies, it has been revealed that cytotoxic T lymphocyte correlated antigen-4 (CTLA-4) is involved in the pathophysiology of MG.^[8–10]^

CTLA-4 is a molecule derived from CTL clone and the CTLA-4 gene co-localizes in the same chromosome as CD28 in human.^[11]^ CTLA-4 is an immunosuppressive receptor on T cells and is an essential negative regulator of T cell response whose mechanism of action is important.^[12,13]^ Studies have proved that CTLA-4 blockade results in T-cell activation, which provides a novel treatment to overcome tumor-induced immune tolerance.^[14,15]^ Other researchers have demonstrated that following T-cell activation, CTLA-4 could be rapidly upregulated and usually binds to B7 molecules, which were higher than CD28.^[16,17]^ Gene methylation plays a key role in detecting and diagnosing cancer in the biopsy specimens and noninvasive body fluids, such as gastric washes and serum.^[18]^ Changes of DNA methylation are heritable and affect X chromosome inactivation, gene expression, parental imprinting, and development.^[19]^ Gene body methylation has been proved to alter gene expression, which is a therapeutic target in the treatment of cancer.^[20]^ A recent discovery on the correlations between CTLA-4′ and MG suggested that CTLA-4 might play a vital role in regulating self-tolerance and affects the susceptibility of autoimmune diseases like MG.^[21]^ In addition, a previous study has shown that acetylcholinesterase (AchE) can help treat MG as it stops the breakdown of acetylcholine, which is found in adhesion with nerves and muscles.^[22]^ IL-8 was also demonstrated to be upregulated in MG.^[23]^ Despite all of the aforementioned findings, very little is known about the role of CTLA-4 methylation in the occurrence of MG. Therefore, the present study was conducted with aims of exploring the effects of CTLA-4 methylation on the pathogenesis of MG and the expression of related cytokines expressions, which might provide some reference in the diagnosis and prognosis of MG in the future.

## 2 Materials and methods

### 2.1 Ethics statement

This study was approved by the ethics committee of Jining No.1 People's Hospital. All of the study participants were provided with a written informed consent. All animal experiments conform to the related animal ethical requirements.

### 2.2 Subjects

From July 1st, 2009 to March 30th, 2015, a total of 103 MG patients (44 females and 59 males) receiving treatment at Jining No.1 People's Hospital were enrolled as the MG group, with a mean age of 41.2 ± 11.0 years (ranging from 6 to 77). The patients selected for the study were only not related and had no family history of MG. The subjects were diagnosed with MG, on the basis of their clinical symptoms, neostigmine test, fatigue test, and repetitive nerve electric stimulation test, with exclusion of other autoimmune diseases. According to the modified classification system, MG patients in the MG group were divided into the ocular myasthenia gravis (OMG) group (n = 39) and the generalized myasthenia gravis (GMG) group (n = 64). Next, they were further allocated into the normal thymus (MN) group (n = 62), the thymic hyperplasia (MH) group (n = 27), and the thymoma (MT) group (n = 14) based on conditions of thymic abnormalities. In total, 106 healthy individuals (54 males and 52 females), within the ages of 5 to 76 years with a mean age of 40.7 ± 10.9 years, who have had their physical examinations done during the same period in Jining No.1 People's Hospital were chosen as the control group. The distribution of gender and age were uniform in both the the control group and the MG group. After all of the subjects went through fasting for 10 to 12 hours, their peripheral venous blood was collected under aseptic conditions in the morning and added into vacuum tubes containing ethylenediaminetetraacetic acid as an anticoagulant for subsequent experiments.

### 2.3 Methylation specific polymerase chain reaction (MSP)

A total of 5 mL peripheral venous blood was extracted from all subjects. Genomic DNA was extracted using a blood genomic DNA Extraction Kit. The ultraviolet spectrophotometer was used to detect the optical density (OD) value at the wavelength of 260 nm, and agarose gel electrophoresis was used to detect its integrity. The DNA template was stored at −20 °C following its purification. The CTLA-4 genes’ CpG island specific methylated (M) and unmethylated (U) primers were designed according to the sequences of CTLA-4 (Gene ID: 1493) (Table 1). All primers were synthesized by Sangon Biotech Co., Ltd. (Shanghai, China). ABI7500 PCR instrument was applied to perform the reverse transcription quantitative polymerase chain reaction (RT-qPCR) amplification of the modified DNA, with SYBR Green I (Roche Diagnostics, Mannheim, Germany) used as the fluorescent marker. The reaction systems of PCR amplification were as follows: 4.8 μL DNA solution, 30 μL 2 × Taq PCR Master Mix, 1.2 μL forward and reverse primers (10 μmol/L), respectively, 22.8 μL double distilled water (DDW), and the total volume was 60 μL. The reaction conditions for RT-qPCR were: pre-denaturation (95 °C) for 5 minutes, 40 cycles of denaturation at 95 °C for 45 seconds, 56 °C for 45 seconds, and 72 °C for 1 minutes, followed by DNA strands extension (72 °C) for 10 minutes. After the PCR cycling reaction, 5 μL products were extracted and loaded onto 2% agarose gel electrophoresis. The gel imaging analysis system was employed for the analysis of the electrophoresis results: methylation specific primers (M) amplified the target band, while the nonmethylation specific primers (U) did not amplify the target band, which was considered as methylated; methylation specific primers (M) did not amplify the target band, while the nonmethylation specific primers (U) amplified the target band, which was considered as not methylated; and 2 pairs of primers amplified the target bands, which were considered as semimethylation, and this condition was also considered to be methylated.

**Table 1:**
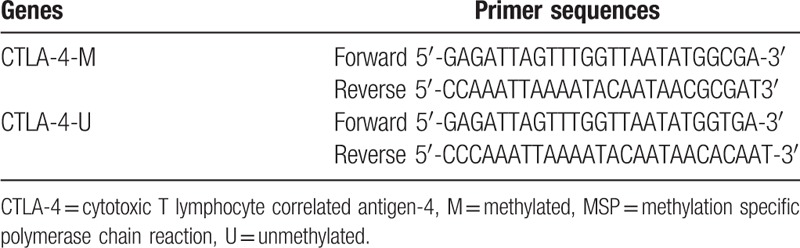
The primer sequences for MSP.

### 2.4 Detection of DNA methyltransferase (DNMT)

Total protein extracted from peripheral blood of the MG and control groups were separately stored at −20 °C. After the protein concentrations of the samples were determined with a bicinchoninic acid (BCA) protein assay kit (ThermoFisher Scientific, San Jose, CA), electrophoresis was performed on 8% gels at 60 V and then transferred to 120 V for 1 to 2 hours. After electrophoresis, the separated proteins were transferred onto the polyvinylidene fluoride (PVDF) membrane for 2 hours at 4 °C. The PVDF membrane was then removed and blocked for 1 to 2 hours with 5% skim milk at room temperature. DNMTl, DNMT3A, DNMT3B, and β-actin (Abcam PLC, Cambridge, UK) was added as the primary antibody and labeled overnight at 4 °C. Next, the samples were rinsed with tris-buffered saline with Tween 20 (TBST) buffer 3 times (10 minutes each time). The 2nd antibody was then added at room temperature was left to sit for 1 hours, followed by rinsing with TBST 3 times (10 minutes each time). The solutions A and B of the chemiluminescence reagent were then mixed evenly on the PVDF membrane at a ratio of 1:1, and the protein bands were developed. All immunoblotted bands were subjected to relative OD analysis.

### 2.5 Reverse transcription quantitative polymerase chain reaction (RT-qPCR)

Total RNA was extracted from the peripheral venous blood of the MG and control groups using the miRNeasy Mini Kit (Qiagen GmbH, Hilden, Germany). This RNA sample (5 μL) was diluted 20 times with RNase-free ultrapure water. The OD value at the wavelength of 260 and 280 nm was read and the concentration and purity of RNA were determined. If a sample had an OD260/OD280 value from 1.7 to 2.1, it was said to have a high purity of RNA, and will have met the requirements for the following experiments. The cDNA template was synthesized through reverse transcription in PCR amplifier, which was performed using ABI7500 quantitative PCR system (Applied Biosystems Inc., Foster City, CA). The reaction conditions were as follows: predenaturation at 95 °C for 5 minutes, 30 cycles of denaturation at 94 °C for 30 seconds, annealing at 55 °C for 30 seconds, extension at 72 °C for 30 seconds, with β-actin used as the internal reference. The primer sequences are shown in Table 2. Each sample was repeatedly detected 3 times. The Opticon Monitor 3 software (Bio-Rad, Inc., Hercules, CA) was applied to analyze the results of RT-PCR. The threshold value was determined at the lowest point of the parallelly rising logarithmic amplification curve in a manual manner, and the (Threshold cycle) Ct values of each reaction tube were obtained. The data were analyzed with 2^−ΔΔCt^ method, and the mRNA expressions of CTLA-4 in the MG and control groups were measured. The experiment was repeated 3 times.

**Table 2:**
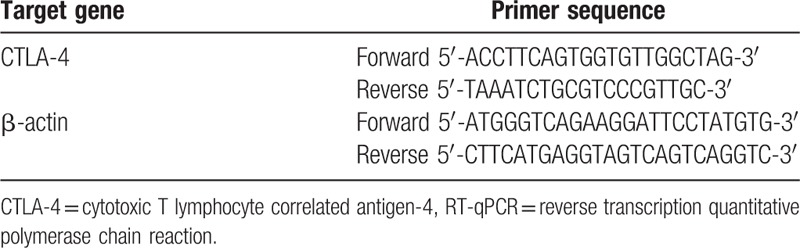
The primer sequences for RT-qPCR.

### 2.6 Establishment of rat model of MG

A total of 45 female Lewis rats (aged 7–8 weeks old with weigh of 140 ± 160 g) (purchased from the Institute of model animals of Nanjing University) were selected for the following study. Next, the rats were fed in the specific-pathogen-free (SPF) animal room under a 12 hours light/night cycle at 22 to 24 °C with a constant humidity of 40% to 70%, and free access to food and water. Ten rats were selected as the adjuvant group, and the remaining 35 rats were selected for experimental autoimmune myastheia gravis (EAMG) model establishment. Thirty model rats were determined and randomly grouped into the EAMG group, the dexamethasone (Tianjin Pharmaceuticals Co., Ltd, the dosage was 0.1 mg/kg/1 time/day and the injection interval was 24 hours) group, the 5-Azad C (DNMT inhibitor, Sigma–Aldrich Chemical Company, Saint Louis, MO, with a dosage of 0.5 μg/μL/kg/1 time/day and an injection interval of 24 hours) group (10 rats in each group). The rats in the dexamethasone group and the NF-AT group were intraperitoneally injected for 8 weeks after the EAMG model was successfully established. The rats in the adjuvant group and the EAMG group were intraperitoneally injected with equal amounts of phosphate buffer saline (PBS). The method of establishing the EAMG model was as follows: 50 μg AchR α97–116 peptide and complete Freund adjuvant (Sigma–Aldrich Chemical Company, St Louis, MO) were mixed fully to obtain the emulsion (200 μL), and then the Lewis rats were subcutaneously injected with the emulsion on their necks, backs, and foot pads. On the 30th day and the 60th day following the first inoculation, 50 μg AchR α97–116 peptide and incomplete Freund adjuvant (Sigma–Aldrich Chemical Company, St Louis, MO) were mixed fully to obtain the emulsion (200 μL) and the enhanced inoculation of the emulsion was performed on the same body parts as the 1st injection. Meanwhile, the rats in the adjuvante group were injected with equal amounts of the emulsion that was prepared using complete/incomplete + normal saline on the same body parts. The EAMG modeling was said to be successful when both the AchR-Ab and the low frequency repetitive electrical stimulation test electrical attenuation were positive, 2 weeks after the last immunization of AchR α97–116 antigen.

### 2.7 Enzyme-linked immunosorbent assay (ELISA)

The concentrations of CTLA-4, acetylcholine receptor antibody (AchR-Ab), titin antibody (Titin-Ab), ryanodine receptor antibody (RyR-Ab), related cytokines such as interleukin-2 (IL-2), interleukin-10 (IL-10), interferon-gamma (IFN-γ), and transforming growth factor β (TGF-β) in the serum samples of the MG and control groups were measured using the ELISA method, according to the manufacturers’ instuctions on the ELISA Kit (Peprotech, Rocky Hill, NJ). The detailed procedures were as follows: the coating buffer was used to dilute the antibody to 1 μg/mL. Next, the antibody was added to 96-well plates (100 μL per well) and was left to be coated overnight at 4 °C. After the coating buffer had been discarded and the plates were rinsed 3 times, each well was supplemented with 150 μL blocking buffer and incubated at room temperature for 1 hours. After plates were rinsed, 100 μL diluted standard samples or serum samples were added, respectively. The blank control group was set and samples were incubated at room temperature for 2 hours. After the plates were rinsed, each well was inserted with 100 μL horseradish peroxidase (HRP)-labeled avidin and incubated for 1 hours at room temperature. The mixture reacted at room temperature for 15 minutes with tetramethyl benzidine (TMB) as a substrate after the plates were washed. Then, 50 μL 2 M sulfuric acid was added to each well with to terminate the reaction. The OD values of each well were recorded with microplate reader at the wavelength of 450 nm, and the concentration (pg/mL) of each cytokine was measured according to the standard curves.

### 2.8 Detection of P-AchE and E-AchE

After the plasma and erythrocytes were obtained through centrifugation (2500 r/minutes, 15 minutes), the blood samples collected were used to determine the activity of plasma acetylcholinesterase (P-AchE) and erythrocyte acetylcholinesterase (E-AchE). One drop of plasma or 1 drop of erythrocyte treated with erythrocyte lysate for 30 minutes was centrifuged (1200 r/minutes, 3 minutes) to get the upper suspension, which was dropped onto dry films with marking enzymes. The Vitro S250 biochemical analyzer (Johnson & Johnson, New Brunswick, NJ) was adopted to measure the activity (U/mL) of P-AchE and E-AchE.

### 2.9 Treg cell ratio detection

A total of 1.5 mL blood was collected from the caudal vein of rats and the rat monocyte-separating medium was used to separate the peripheral blood mononuclear cells (PBMC) (mainly containing monocytes and lymphocytes) at the density of 1.082 g/L. The cells were then washed with PBS 3 times and counted, followed by the adjustement of the cell concentration to 10^6^/mL and inoculation to a 12-well plate (1 mL each well). The monocytes with easy adhesions to the wall were separated from the lymphpcytes, which were suspended. The lymphocytes were then selected to be cultured in vitro for 3 days stimulated by R-ACh Rα 97–116 peptide, with the FACS method applied to examine the difference of CD4+ T cell subtype. Before staining, the cell culture fluid was incubated with BFA (diluted at 1:1), PMA (20 ng/mL) and Inomycin (1 μg/mL) for 4 hours. The cells were then stained by FITC-anti-rat CD4, fixed to rupture the membranes, added with the primary antibody PE-antirat Foxp3 secondary antibody PE-Cy3 antirabbit Ig G and lastly examined by flow cytometry.

### 2.10 Statistical analysis

All data were analyzed with SPSS 21.0 statistical software (SPSS, Inc, Chicago, IL). The measurement data were expressed by mean ± standard deviation. Variance analysis was performed in comparison among multiple groups and the LSD-t was applied in pairwise comparison between 2 groups. Frequency and frequency rate were adopted to express the count data. Chi-square test was used to analyze categorical data. Rank sum test was applied to compare ordinal data. *P* < .05 indicates statistically significant differences.

## 3 Results

### 3.1 MG patients had a higher methylation incidence

The results from the experiments performed to verify the incidence of CTLA-4 methylation are shown in Table 3. The proportion of methylation in the promoter region of CTLA-4 gene in peripheral blood was 19.81% (21/106) in the control group, while it was 33.98% (35/103) in the MG group. Therefore, the methylation incidence in the promoter region of CTLA-4 gene in the MG group was significantly higher than that in the control group (*P* < .05).

**Table 3:**
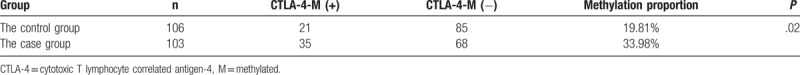
Methylation in the promoter region of CTLA-4 in peripheral blood of the case and control groups.

### 3.2 CTLA-4 methylation was related to thymus status

The relations between CTLA-4 methylation and the thymus status of MG patients were detected in the above experiment and the association of CTLA-4 methylation with clinicopathological characteristics of MG patients results were as shown in Table 4. There was no significant difference in CTLA-4 methylation in regard to age stratification, gender distribution, MG type, concurrent hyperthyroidism, and prior history of MG (*P* > .05). However, the CTLA-4 methylation was significantly correlated with thymus status (*P* < .05).

**Table 4:**
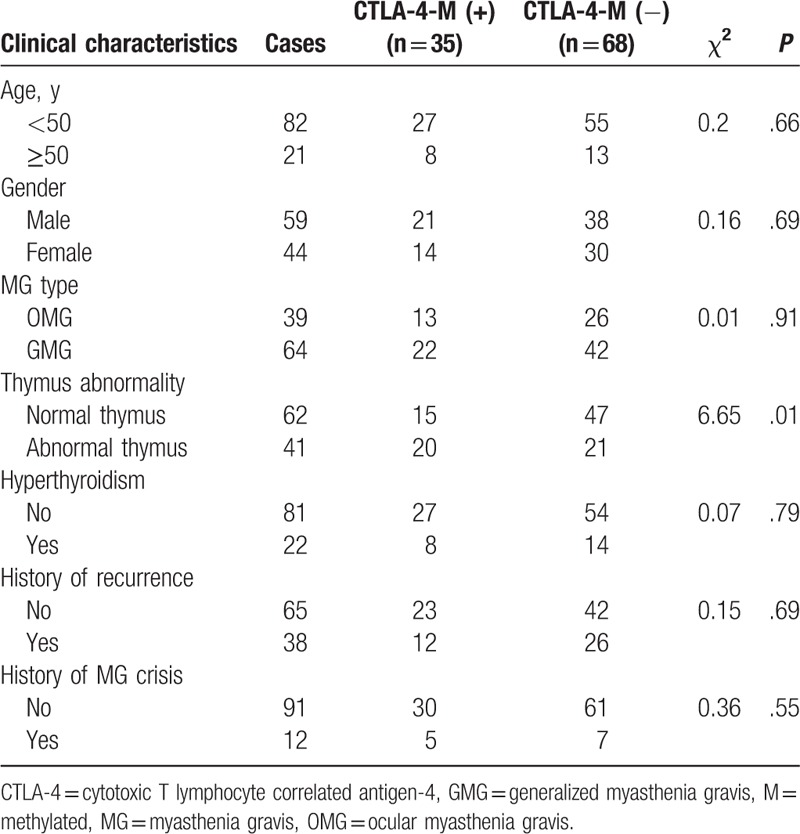
The correlation between CTLA-4 methylation and clinicopathological features of MG patients.

### 3.3 MG patients had increased the expressions of DNMTl, DNMT3A, and DNMT3B

**Figure 1:**
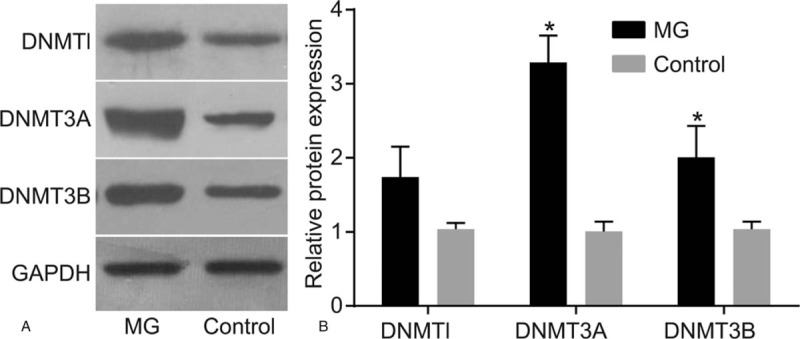
MG patients increased the expressions of DNMTl, DNMT3A, and DNMT3B. Notes: Data were compared by independent sample *t* test. ^∗^*P* < .05 compared with the control group; the MG group (n = 103). Control, the negative control group (n = 106); DNMT = DNA methyltransferase, MG = myasthenia gravis.

Western blot analysis was performed in order to detect the expressions of DNMTs and the results obtained, as shown in Fig. 1 indicated that the expressions of DNMTl, DNMT3A, and DNMT3B in the MG group were significantly higher (*P* < .05) when compared with the control group.

### 3.4 MG patients had decreased mRNA expression of CTLA-4

**Figure 2:**
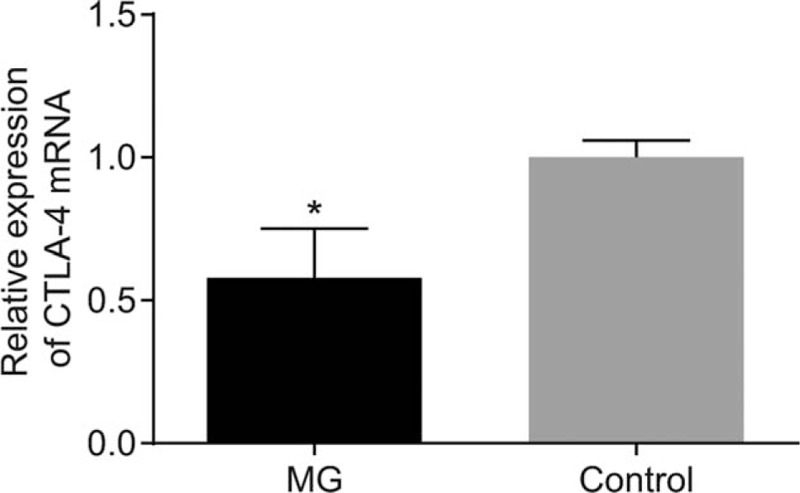
MG patients increased the mRNA expression of CTLA-4. Notes: Data were compared by independent sample *t* test. ^∗^*P* < .05 compared with the control group; the MG group (n = 103). Control, the negative control group (n = 106); CTLA-4 = cytotoxic T lymphocyte correlated antigen-4, MG = myasthenia gravis.

RT-qPCR was applied to examine the mRNA expression of CTLA-4 in 2 groups. The data accumulated showed that the expressions of CTLA-4 mRNA in the control and MG groups were 1.00 ± 0.06 and 0.58 ± 0.17, respectively (Fig. 2), thus indicating that the expression of CTLA-4 is lower in the MG group in comparison with the control group (*P* < .05).

### 3.5 MG patients had higher expressions of AchR-Ab, titin-Ab and RyR-Ab and IL-2, IL-10, IFN-γ, and TGF-β and lower expression of CTLA-4

**Figure 3:**
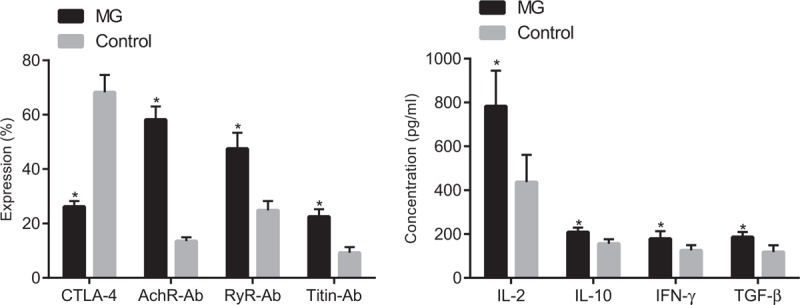
MG patients increased expressions of AchR-Ab, titin-Ab and RyR-Ab and IL-2, IL-10, IFN-γ, and TGF-β and decreased expression of CTLA-4. Notes: Panel A, the expressions of CTLA-4, AchR-Ab, titin-Ab, and RyR-Ab in peripheral blood; Panel B, expressions of IL-2, IL-10, IFN-γ, and TGF-β. Data were compared by independent sample *t* test.^∗^*P* < .05 compared with the control group; the MG group (n = 103). Control, the negative control group (n = 106). CTLA-4 = cytotoxic T lymphocyte correlated antigen-4, IFN-γ = interferon-gamma, IL-2 = interleukin-2, IL-10 = interleukin-10, MG = myasthenia gravis, TGF-β = transforming growth factor β.

The results from ELISA, which was employed to detect the expressions of serum-related factors and concentration of cytokines, indicated (Fig. 3) that the expressions of serum AchR-Ab, titin-Ab, and RyR-Ab in the MG group were significantly higher than those in the control group, while the expression of serum CTLA-4 was reduced (*P* < .05). The expressions of IL-2, IL-10, IFN-γ, and TGF-β were also higher in the MG group when compared with those in the control group (*P* < .05).

### 3.6 MG patients had higher activities of P-AchE and E-AchE

**Figure 4:**
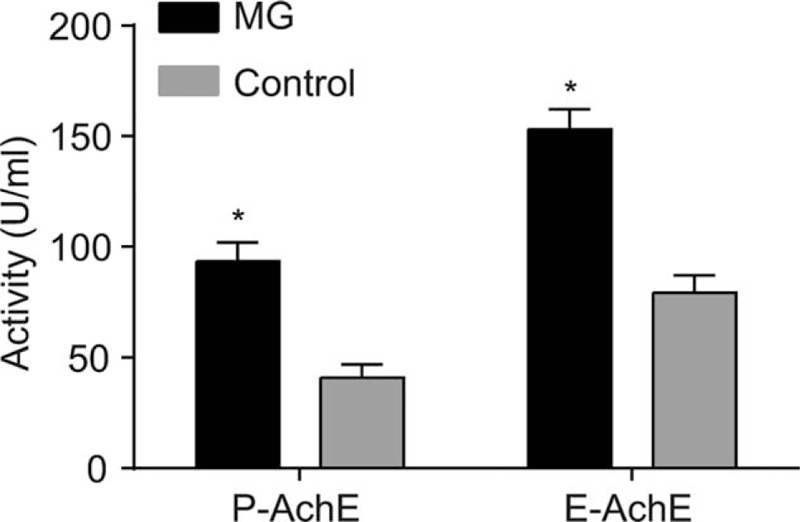
MG patients increased the activities of P-AchE and E-AchE. Notes: Data were compared by independent sample *t* test. ^∗^*P* < .05 compared with the control group; MG, myasthenia gravis, the MG group (n = 103). Control, the negative control group (n = 106). E-AchE = erythrocyte acetylcholinesterase, MG = myasthenia gravis, P-AchE = plasma acetylcholinesterase.

The activities of P-AchE and E-AchE in the MG and control groups were detected. The results were as follows: the activities of P-AchE in the control and MG groups were 40.89 ± 6.06 and 93.26 ± 8.65 U/mL, respectively. The activities of E-AchE in the control and MG groups were 79.35 ± 7.94 and 153.03 ± 9.05 U/mL, respectively. These findings highly indicated that compared with the control group, the activities of P-AchE and E-AchE were markedly higher in the MG group (*P* < .05) (Fig. 4).

### 3.7 CTLA-4 methylation has correlations with the expressions of CTLA-4, AchR-Ab, Titin-Ab, RyR-Ab, P-AchE, E-AchE, IL-2, IL-10, IFN-γ, and TGF-β

Based on the results from the statistical analysis on the correlations of CTLA-4 methylation with the expressions of related cytokines in MG patients, CTLA-4 methylation was found to be closely correlated with CTLA-4 serum levels and expressions of AchR-Ab, Titin-Ab, RyR-Ab, P-AchE, E-AchE, IL-2, IL-10, IFN-γ, and TGF-β (*P* < .05) (Table 5).

**Table 5:**
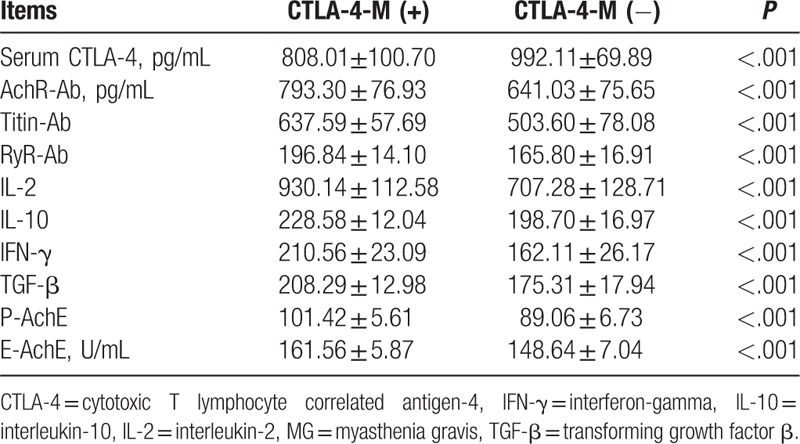
The correlation of CTLA-4 methylation and expressions of related cytokines in MG patients.

### 3.8 CTLA-4 methylation leads to the suppression of AchR-Ab expression, the activity of E-AchE and expressions of IL-2, IL-10, IFN-γ, and TGF-β

**Figure 5:**
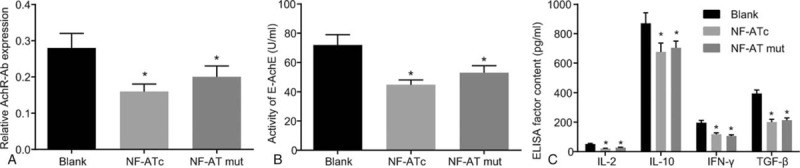
The CTLA-4 methylation suppressed the expression of AchR-Ab, the activity of E-AchE and expressions of IL-2, IL-10, IFN-γ, and TGF-β. Notes: Panel A, expression of AchR-Ab; Panel B, activity of E-AchE; Panel C, expressions of IL-2, IL-10, IFN-γ, and TGF-β. Data were compared by ANOVA. ^∗^*P* < .05 compared with the blank group. The experiment was repeated 3 times. ANOVA = one-way analysis of variance, CTLA-4 = cytotoxic T lymphocyte correlated antigen-4, E-AchE = erythrocyte acetylcholinesterase, IL-2 = interleukin-2, IL-10 = interleukin-10, IFN-γ = interferon-gamma, TGF-β = transforming growth factor β.

In order to verify the effect of CTLA-4 methylation, small amount of thymus tissues were collected aseptically and cut, followed by the filtration of the tissues through 80 stainless steel mesh to prepare single cell suspension, which was to separate the thymocyte through a lymphocyte separation medium. After being treated with NF-ATc and NF-AT mut (NF-AT treatment, which affected the upstream starting site of the CTLA-4 promoter region and interfered with its methylation, 50 nmol/mL), the untreated cells were used as control (the blank group). The results of expressions of cytokines examined by ELISA are shown in Fig. 5. The expression of AchR-Ab, the activity of E-AchE and expressions of IL-2, IL-10, IFN-γ, and TGF-β were higher (*P* < .05), while the expression of AchR-Ab, the activity of E-AchE and expressions of IL-2, IL-10, IFN-γ, and TGF-β were inhibited in the NF-ATc and NF-AT mut groups (*P* < .05).

### 3.9 The dexamethasone and 5-Azad C groups inhibited the expression of AchR-Ab, the activity of E-AchE and expressions of IL-2, IL-10, IFN-γ, and TGF-β

**Figure 6:**
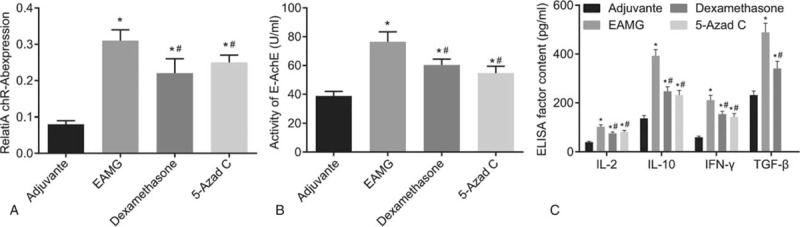
The dexamethasone and 5-Azad C groups inhibited the expression of AchR-Ab, the activity of E-AchE and expressions of IL-2, IL-10, IFN-γ, and TGF-β. Notes: Panel A, expression of AchR-Ab; Panel B, activity of E-AchE; Panel C, expressions of IL-2, IL-10, IFN-γ, and TGF-β. ANOVA was used to compare. ^∗^*P* < .05 compared with the NC group; ^#^*P* < .05 compared with the EAMG group (n = 10). The experiment was repeated 3 times. ANOVA = one-way analysis of variance, E-AchE = erythrocyte acetylcholinesterase, EAMG = experimental autoimmune myastheia gravis, IFN-γ = interferon-gamma, IL-2 = interleukin-2, IL-10 = interleukin-10, TGF-β = transforming growth factor.

The effect of the inhibition of CTLA-4 methylation on serum-related cytokines of MG rats were further examined in vivo experiments. The results showed that 8 weeks after administration, the rats in the EAMG group had higher AchR-Ab expression, E-AchE activity and IL-2, IL-10, IFN-γ, and TGF-β expressions (*P* < .05), while the expression of AchR-Ab, the activity of E-AchE, and expressions of IL-2, IL-10, IFN-γ, and TGF-β were all inhibited in rats in the dexamethasone and the 5-Azad C groups (*P* < .05) (Fig. 6).

### 3.10 The dexamethasone and 5-Azad C groups inhibited the Treg cell ratio in lymphocytes

**Figure 7:**
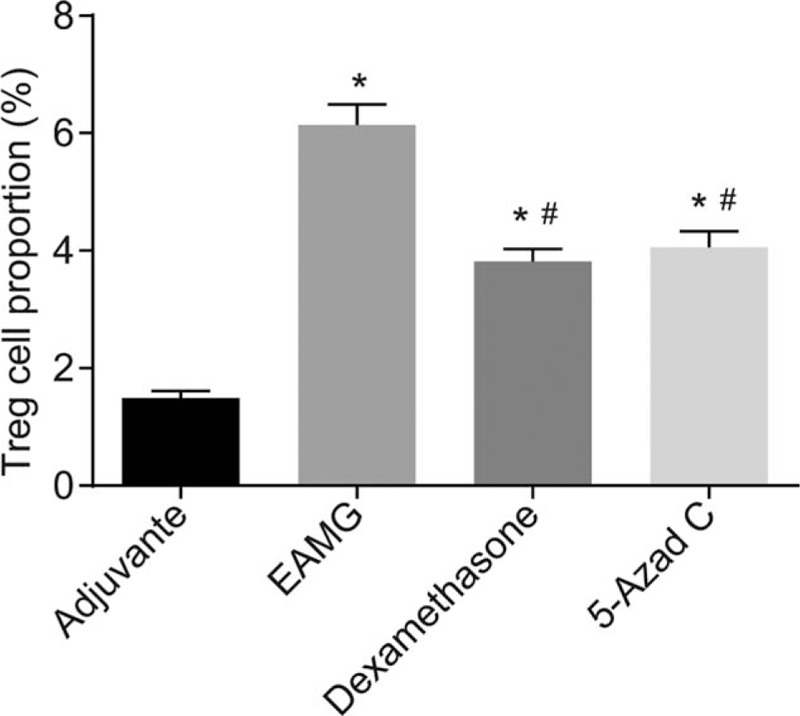
The dexamethasone and 5-Azad C groups inhibited the Treg cell ratio in lymphocytes. Notes: ANOVA was used to compare. ^∗^*P* < .05 compared with the NC group; ^#^*P* < .05 compared with the EAMG group (n = 10). The experiment as repeated 3 times. ANOVA = one-way analysis of variance, EAMG = experimental autoimmune myastheia gravis, NC = negative control.

Flow cytometry was employed to evaluate the effect of inhibiting CTLA-4 methylation on Treg cell ratio. The results, as shown in Fig. 7, indicated that the rats in the EAMG group had higher Treg cell ratio in lymphocytes (*P* < .05), while rats in the dexamethasone group and the 5-Azad C group significantly inhibited the Treg cell ratio in lymphocytes (*P* < .05) 8 weeks after administration.

## 4 Discussion

MG is a severe autoimmune disease that occurs as a result of a decrease in efficiency of synaptic transmission at neuromuscular synapses.^[24]^ Methylation is involved in the epigenetic regulation of X chromosome inactivation, gene expression, genomic imprinting, and development.^[25]^ DNA methylation helps regulate the expression of CTLA-4, which is an inhibitory molecule that is of significance in the regulation of T-reg cell function.^[26]^ The present study was conducted with aims of broadening the understanding on the effects of CTLA-4 methylation on the pathogenesis and expressions of related cytokines in MG patients.

Our initial finding presented indicated that the incidence of CTLA-4 methylation in the MG group was significantly higher than that in the control group, suggesting that CTLA-4 methylation was correlated with MG. This result was also consistent with a previous study showing that CTLA-4 gene polymorphisms could affect the expression of the protein and thus influence the immune response, and the single nucleotide polymorphism (SNP) at position +49 in the coding sequence (CDS) 1 of the CTLA-4 gene, which had been proven to be correlated with MG.^[27]^ A number of other studies have also revealed that CTLA-4 might inhibit the immune response and also be involved in the pathophysiology of MG.^[9,10]^ It has also been found that the (AT) n of CTLA-4 could facilitate mutation through inducing T cell reactivity through the CD28 pathway, and the (AT)n polymorphism might contribute to the decreased mRNA stability, leading up to reduced CTLA-4 expression in MG patients.^[28,29]^ These findings helped to confirm the attached importance on CTLA-4 methylation in the pathophysiology of MG.

In addition, this study also revealed that when compared with the control group, the expressions of DNMTl, DNMT3A, and DNMT3B were significantly higher in the MG group (*P* < .05). Based on previous findings, there are mainly 3 catalytic active DNMT in mammals: DNMT1, DNMT3A, and DNMT3B; DNMT1 is responsible for maintaining the DNA methylation over replication, and its deletion might result in rapid cell death in the human embryonic stem cells.^[30,31]^ DNMT3A is required for the methylation of imprinted genes and the dispersed repeated elements like retrotransposons, and DNMT3B plays a key role in the methylation of the pericentric satellite repeats.^[32]^ As they are methyltransferases, DNMT3A and DNMT3B are required in the establishment of the genomic methylation patterns in the embryonic development.^[33]^ Moreover, Challen et al suggested that DNMT3A and DNMT3B have distinct functions in the hematopoietic stem cells which ensure the lifelong production of bone marrow and blood.^[34]^ Another study has also indicated that DNMT3B might play a role in the promotion of thymoma in MG patients.^[34]^ Based on the results from the present study, there were significantly higher levels of DNMTl, DNMT3A, and DNMT3B expressions higher in the MG patients, which is consistent with the aforementioned findings.

Furthermore, our study demonstrated that, when compared with the control group, both the expressions of AchR-Ab, titin-Ab, RyR-Ab, IL-2, IL-10, IFN-γ, and TGF-β and the activity of P-AchE and E-AchE were higher while the expression of CTLA-4 was lower in the MG group (*P* < .05), indicating the correlation between MG and the expressions of CTLA-4, AchR-Ab, Titin-Ab, RyR-Ab, IL-2, IL-10, IFN-γ, and TGF-β. Based on a study by Verschuuren et al, it has been proved that MG is caused by antibodies which interfere with the function of postsynaptic proteins including complement activation, competition with ligand binding sites, and antigenic modulation.^[35]^ By increasing their turnover and degradation, these antibodies can reduce the number of functional acetylcholine receptors (AChRs).^[36]^ It has been reported that the antibodies involved in the pathogenetic role in MG patients includes AchR-Ab, titin-Ab, and presynaptic membrane antibody (RyR-Ab).^[37]^ Thus, the reduction of anti-AchR-Ab can be used in the treatment of MG.^[38]^ A previous study revealed that reduced expressions of acetylcholine receptor-specific IL-2, IL-10, and IFN-γ is closely linked to the occurrence of MG.^[39]^ In addition, the expression of IL-10 had a negative correlations with let-7c expression in the peripheral blood mononuclear cells of MG patients.^[40]^ We have come across another study with a key finding suggesting that bone marrow stromal cells might ameliorate MG via altering the balance of Treg cell subsets through TGF-β secretion.^[41]^ Therefore, these studies have made it clear that MG was closely correlated with the expressions of CTLA-4, AchR-Ab, Titin-Ab, RyR-Ab, IL-2, IL-10, IFN-γ, and TGF-β.

In conclusion, the present study explored the regulatory effects of CTLA-4 methylation in the pathogenesis of MG and the expression of related cytokines. The role of CTLA-4 methylation in the occurrence of MG was further investigated and the findings revealed that CTLA-4 methylation could in fact influence the occurrence of MG and related cytokines expressions in MG patients by regulating AchR-Ab and E-AchE. Moreover, the present study provided evidence on the correlations between CTLA-4 methylation and the pathogenesis of MG. However, due to the small sample size and time limit, more studies are required to further clarify the specific underlying mechanism in the regulation AchR-Ab and E-Ach activities by CTLA-4 methylation.

## Acknowledgment

The authors thank their appreciation to reviewers for their helpful comments.

## Author contributions

**Conceptualization:** Ti-Kun Fang, Cheng-Jun Yan, Juan Du.

**Data curation:** Juan Du.

**Formal analysis:** Ti-Kun Fang, Cheng-Jun Yan, Juan Du.

**Investigation:** Ti-Kun Fang, Cheng-Jun Yan, Juan Du.

**Methodology:** Ti-Kun Fang, Cheng-Jun Yan.

**Project administration:** Ti-Kun Fang, Cheng-Jun Yan, Juan Du.

**Resources:** Ti-Kun Fang, Cheng-Jun Yan.

**Software:** Juan Du.

**Supervision:** Ti-Kun Fang, Cheng-Jun Yan.

**Validation:** Ti-Kun Fang, Cheng-Jun Yan, Juan Du.

**Visualization:** Ti-Kun Fang, Cheng-Jun Yan, Juan Du.

**Writing – original draft:** Ti-Kun Fang, Cheng-Jun Yan.

**Writing – review & editing:** Ti-Kun Fang, Cheng-Jun Yan, Juan Du.
